# Observations on Mr. Smith's Theory of Sensation

**Published:** 1811-10

**Authors:** James Woodham

**Affiliations:** Blackfriars


### *To the Editors of the Medical and Physical Journal.*

"The motions of life are not more inexplicable than that of bodies towards each other, as in the gravitation of the solar system; yet, in this case, we do not call in the aid of a *fluid* to render the motion more complicated, but wisely confine ourselves to a statement of the *fact.* We are conscious of certain feelings, and we have uniformly found some of these feelings succeeded by muscular motion; but though, by the future improvement of our optical instruments, a subtile fluid were shewn to us, and its vibrations, or vibratiuncles, or direct motion pointed out; we should, indeed, have traced another *link* in the series of *changes*, but we should not be justified in regarding the *motions* of the *fluid* as constituting our *feelings*." BROWN.

GENTLEMEN,

I HAVE been induced to trouble you with the above quotation from a truly philosophical writer, as well as to subjoin the following remarks, in consequence of a very ingenious paper which appeared in your number for June last, entitled "Theory of Sensation," by Mr. Smith of Bristol.

The author commences his essay with objecting to the common, as well as to the Darwinian theory of sensation; he then states his own, *viz.* "Sensation is felt when an action attempted by the vital power is, in any degree, obstructed or interrupted; or, in other words, when an action performed is less than the power exerted;" and after producing his proof says toward the conclusion, "it is not difficult to conceive that an active intelligent power should *feel* when its motions are interrupted." I would, therefore, beg leave to ask the ingenious writer, as he has not defined it, what he understands by the term vital power? Does he understand by it the nervous fluid of Cullen, or the spirit of animation of Darwin? And if either of these, in what sense has it any claim to be considered as *intelligent?* Mr. Smith's theory appears to me to labour under the same defect as that of his once celebrated predecessor; for granting the existence of this fluid or spirit, (at present a mere hypothesis) it would, I conceive, be highly unphilosophical to call it *intelligent*, or the obstruction or

 interruption interruption of its motions *sensation*. Intelligence is mind, and sensation a mental affection, *consequent* to the impression of a body on an organ of sense; and however mysterious or incomprehensible be that "subtle feeling which elevates us to the rank of gods, or degrades us below the dull insensibility of the earth on which we tread," it is not, I think, accordant with the principles of sound philosophy, though it has the sanction of modern physiology*, to ascribe it to a system of organized matter.

I am, &c,

JAMES WOODHAM.

*Blackfriars, August 28th,* 1811.

* "Le cerveau-c'est le centre sensitif qui voit les couleurs, *entend* les sons, &c. * * * * * * * * * *

Le cerveau agit sur les impressions que les nerfs lui transmettent, comme l'œsophage sur les alimens que l'œsophage y verre; *il les digère à sa maniere*, ebranlé par le mouvement qui lui est communiqué, il réagit, et de cette réaction naît la *sensation perceptive* ou la perception."

RICHERAND.

"The *impression* produced on any organ by the action of an external body, constitutes *sensation*. This sensation, transmitted by the nerves to the brain, is *perceived*, that is *felt* by the *organ* (the brain), the sensation then becomes *perception*."

"An *idea* is only a sensation *transformed* or *perceived* by the *cerebral organ*." HOOPER.

